# Multiple Cemento-Ossifying Fibroma: A Sign of Hyperparathyroidism-Jaw Tumour Syndrome

**DOI:** 10.1155/2023/4664619

**Published:** 2023-03-08

**Authors:** Nurul Inaas Mahamad Apandi, Nor Nazaliza Basri, Ajura Abdul Jalil, Md Arad Jelon

**Affiliations:** ^1^Department of Diagnostics and Craniofacial Biosciences, Faculty of Dentistry, The National University of Malaysia, Kuala Lumpur, Malaysia; ^2^Oral Pathology and Oral Medicine Unit, Department of Oral & Maxillofacial Surgery, Kuala Lumpur Hospital, Ministry of Health, Kuala Lumpur, Malaysia; ^3^Stomatology Unit, National Institute of Health, Ministry of Health, Setia Alam, 40170 Shah Alam, Selangor, Malaysia; ^4^Oral and Maxillofacial Surgery Unit, Department of Oral & Maxillofacial Surgery, Kuala Lumpur Hospital, Ministry of Health, Kuala Lumpur, Malaysia

## Abstract

Hyperparathyroidism-jaw tumour (HPT-JT) syndrome is a rare autosomal dominant disease. It is caused by a gene mutation of the tumour suppressor gene CDC73 that encodes for parafibromin. This syndrome predisposes to a triad occurrence of multiple maxillary or mandibular cemento-ossifying fibroma, parathyroid adenoma or carcinoma, and renal and uterine tumours. In this study, we report a case of HPT-JT occurring in a 30-year-old male patient.

## 1. Introduction

Primary hyperparathyroidism (PHPT) is a sporadic disorder with a familial hereditary tendency. It can occur by itself or as part of a syndrome with involvement of tumours in other tissues. Spectrum of the condition includes multiple endocrine neoplasia types 1 (MEN1) and 2A (MEN2A), hyperparathyroidism-jaw tumour (HPT-JT) syndrome, familial benign hypercalcemia, and familial isolated hyperparathyroidism (FIHP) [1].

HPT-JT is a rare autosomal dominant disease. It is an inherited endocrine neoplastic syndrome, which predisposes to a triad occurrence of multiple maxillary or mandibular cemento-ossifying fibroma, parathyroid adenoma, or carcinoma with the presence of renal and uterine tumours as well [2]. The disease is caused by gene mutation of the tumour suppressor gene CDC73 or now known as HRPT2, which encodes for parafibromin [3]. HPT-JT has a higher association prevalence with atypical adenomas and carcinoma compared with other PHPT conditions [4]. Clinical presentation of the disease includes jaw swelling causing disfigurement and related tooth abnormalities secondary to ossifying fibroma. Blood serum may reveal signs of hypercalcemia secondary to hyperparathyroidism due to an underlying benign or malignant tumours. Here, we report a case of HPT-JT occurring in a 30-year-old male patient.

### 1.1. Case Presentation

A 30-year-old man presented to the Oral and Maxillofacial Clinic in Hospital Kuala Lumpur, Kuala Lumpur, Malaysia, with swelling of the right facial region. Upon history, the swelling had been present since 2016 and gradually increased in size. Multiple biopsies were also done. Medically, the patient has underlying hypertension secondary to hypercalcemia. Patient also is a known case of Brown's tumour secondary to hyperparathyroidism and has had a history of parathyroid gland removal in 2014 done in Hospital Putrajaya.

Orthopantomography (OPG) in 2016 showed the presence of a well-defined unilocular radiolucency extending from the apex of the right lower premolar tooth (#44) to the angle of the mandible with a sclerotic border. Root resorption of lower right posterior teeth (#44, #45, #46, and #47) was observed. An incisional biopsy was taken. However, it was inadequate for a definitive diagnosis. In 2018, a cone-beam computed tomography was done and the results suggested malignancy. Thus, again an incisional biopsy was done and a diagnosis of benign fibro-osseous lesion was considered. In 2019, the swelling persisted and the patient presented with an ulceroproliferative growth at the right vestibule opposite the lower right premolar tooth (#45) extending distally. It was covered in slough and the tooth was mobile. Again, an incisional biopsy was performed and a diagnosis of benign fibro-osseous lesion suggestive of cemento-ossifying fibroma was established. However, patient defaulted appointments and only came back in 2021.

**Figure 1:**
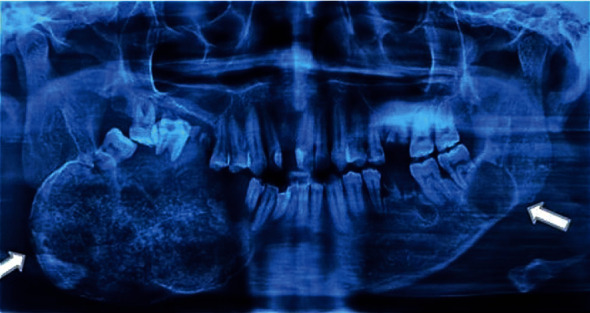
OPG shows presence of multiple well-defined radiolucent with admixed radiopacity on the right body of the mandible. With a unilocular radiolucency on the left body of mandible. Thinning of the cortex is also observed.

Extra-orally, there is a diffuse, firm, and hard swelling on the right side of the mandible. Patient's mouth opening was also limited. Intra-orally, an exophytic growth was evident at the right lower quadrant region causing displacement of the dentition. Another soft tissue lesion at the left third molar region (tooth #38) was also noted. OPG showed the presence of a multilocular radiolucency over the right mandible region along with a unilocular radiolucency on the left mandibular region (Figure 1). Hence, the patient was scheduled for tumour resection on the right mandible and incisional biopsy of the lesion at the tooth #38 region. These specimens were submitted for further histopathological examination.

**Figure 2:**
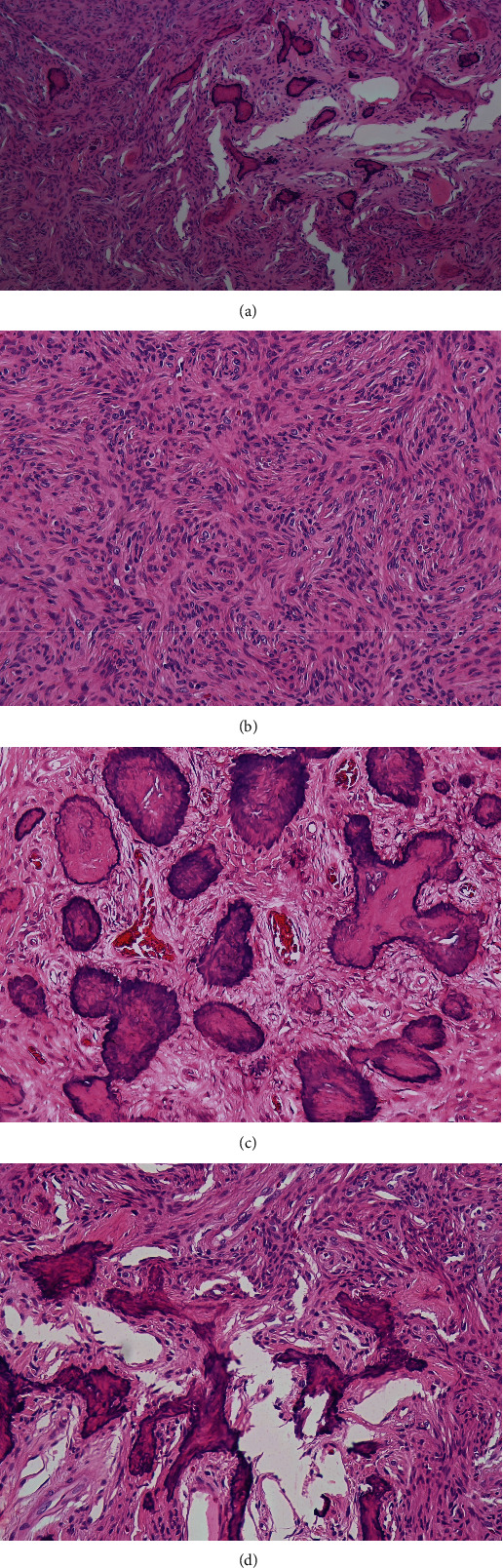
Photomicrograph shows (a) fibrocellular tissue and scattered mineralized material of varying appearances (H&E, 40×); (b) hypercellular fibrous tissue consists of cytologically bland spindle to ovoid fibroblasts arranged in a storiform pattern (H&E, 200×); (c) fused globules of cementum-like material with occasional brush borders (H&E, 200×); and (d) similar histological features of the specimen at the tooth #38 region (H&E, 200×).

Microscopically, the hematoxylin and eosin (H&E) stained sections revealed a well-demarcated lesion composed of fibrocellular tissue and scattered mineralized material of varying appearances (Figure 2(a)). The hypercellular fibrous tissue consists of cytologically bland spindle to ovoid fibroblasts arranged in a storiform pattern (Figure 2(b)). Components were predominantly discrete or fused globules of cementum-like material with occasional brush borders. Irregular trabeculae of woven were also evident (Figure 2(c)). The specimen at the tooth #38 region also showed similar features to the resected tumour specimen (Figure 2(d)). Therefore, a definitive diagnosis of cemento-ossifying fibroma was established for both lesions.

## 2. Discussion

The onset of PHPT in HPT-JT patients occurs at a relatively young age, which is below 20 years and is a typical main finding [5]. Earliest age of hypercalcemia was reported at 7 years old [6]. While sporadic, PHPT presents at above 30 years of age [5]. This is seen in our case, whereby the patient currently suffers from underlying hypertension, which developed secondary to his hypercalcemia condition.

Studies have shown that 25–50% of HPT-JT patients develop cemento-ossifying fibroma of the jaws and favour the mandible over the maxilla [7, 8]. Ossifying fibroma has been categorized as a benign fibro-osseous neoplasm by the World Health Organization and histologically is composed of a fibrocellular stroma with the presence of calcified material [9]. These lesions generally occur within the thirrd to fourth decades of life; however, in HPT-JT patients occur earlier within the second decade of life [10]. The jaw lesion may precede hypercalcemia development by years; thus, leading to misdiagnosis [11]. For our patient, this differs slightly as the patient was diagnosed with hypercalcemia prior to noticing the jaw lesion that developed in 2016 when the patient was in his 30's and persisted until now. However, to caution that the jaw lesion may have already been present and was asymptomatic before becoming noticeable.

A study suggested that the jaw tumours in HPT-JT patients is not caused directly by the hyperparathyroidism; but, instead is related to the gene mutation in HRPT2. This may be the reason, whereby this tumour does not regress even after parathyroidectomy and correction of patients hypercalcemic status [7]. This is seen in our patient, as even with a history of parathyroid gland removal in 2014, the lesion did not reduce in size; however, persisted with involvement of multiple quadrants.

Renal involvement in HPT-JT is limited with renal cyst being the most common manifestation. Otherwise, some patients may develop hamartomas and rare renal tumours, such as adult Wilms' tumour and mixed epithelial–stromal tumours. Wilms' tumours in HPT-JT has displayed different features, such as smaller sized compared with childhood type, poorly circumscribed, lacking necrosis, as well as haemorrhage with low mitotic rate and do not metastasize [8]. In our case, the patient was only noted to have underlying renal atrophy and was under review.

In HPT-JT, management of cemento-ossifying fibromas requires complete surgical resection of the jaw along with reconstruction and grafting of bone. Those suffering from parathyroid lesions are subjected to subtotal or total parathyroidectomy with reference to the level of hormones and parathyroid disease severity [12]. Evaluation of the kidney and uterus status can be done with imaging via ultrasound (US) or magnetic resonance imaging. To date, there is no definitive surveillance guide for HPT-JT patients; however, a study suggested that carriers of the HRPT2 gene should undergo biannual screening of serum calcium and parathyroid hormone (PTH) with periodic US of the parathyroid, five-yearly panoramic dental imaging, five-yearly renal lesion monitoring, and imaging starting at age of diagnosis, and female patients to undergo regular gynaecology examinations and consultation beginning at the reproductive age [13].

## 3. Conclusion

Dental healthcare providers should take into consideration the possibility of the diagnosis of HPT-JT in patients who present with multiple cemento-ossifying fibroma and PHPT, especially those at a young age. A thorough history taking and clinico-radiological examination are crucial prior to the management of these patients. Early diagnosis of HPT-JT may help in identifying and treating other tumours associated with HPT-JT. HPT-JT patients with jaw tumours should need close monitoring due to the high-risk potential of recurrence and occurrence of parathyroid malignancies.
